# Absorption of Manganese and Iron in a Mouse Model of Hemochromatosis

**DOI:** 10.1371/journal.pone.0064944

**Published:** 2013-05-21

**Authors:** Jonghan Kim, Peter D. Buckett, Marianne Wessling-Resnick

**Affiliations:** 1 Department of Pharmaceutical Sciences, Northeastern University, Boston, Massachusetts, United States of America; 2 Department of Genetics and Complex Diseases, Harvard School of Public Health, Boston, Massachusetts, United States of America; Penn State Hershey Medical Center, United States of America

## Abstract

Hereditary hemochromatosis, an iron overload disease associated with excessive intestinal iron absorption, is commonly caused by loss of HFE gene function. Both iron and manganese absorption are regulated by iron status, but the relationships between the transport pathways of these metals and how they are affected by HFE-associated hemochromatosis remain poorly understood. Loss of HFE function is known to alter the intestinal expression of DMT1 (divalent metal transporter-1) and Fpn (ferroportin), transporters that have been implicated in absorption of both iron and manganese. Although the influence of HFE deficiency on dietary iron absorption has been characterized, potential effects on manganese metabolism have yet to be explored. To investigate the role of HFE in manganese absorption, we characterized the uptake and distribution of the metal in *Hfe* ^−/−^ knockout mice after intravenous, intragastric, and intranasal administration of ^54^Mn. These values were compared to intravenous and intragastric administration of ^59^Fe. Intestinal absorption of ^59^Fe was increased and clearance of injected ^59^Fe was also increased in *Hfe^−/−^* mice compared to controls. *Hfe* ^−/−^ mice displayed greater intestinal absorption of ^54^Mn compared to wild-type *Hfe^+/+^* control mice. After intravenous injection, the distribution of ^59^Fe to heart and liver was greater in *Hfe* ^−/−^ mice but no remarkable differences were observed for ^54^Mn. Although olfactory absorption of ^54^Mn into blood was unchanged in *Hfe* ^−/−^ mice, higher levels of intranasally-instilled ^54^Mn were associated with *Hfe^−/−^* brain compared to controls. These results show that manganese transport and metabolism can be modified by HFE deficiency.

## Introduction

High levels of manganese impair neurobehavior in both humans and animal models [1]–[5]. Fortunately, manganese loading due to ingestion is relatively rare since hepatic first-pass elimination of the metal provides a protective mechanism against toxicity [6]. However, intake of airborne manganese bypasses the biliary excretion route and inhaled manganese is efficiently transported into the body including the brain through the nasal epithelium [7]–[9]. High levels of airborne manganese are common in occupational settings of mining, manganese ore processing, dry battery manufacture and organochemical fungicide use [10], [11], raising concerns about public and occupational health problems. Recent work by Haynes et al. [12] determined hair and blood manganese levels in residents living near Marietta OH and a ferromanganese refinery that is a major US airborne emission source. The relationship between these biomarkers and ambient air levels of manganese became significant when iron metabolism genes, including *HFE* (hyperferremia) alleles, were incorporated in their models [12]. Our group recently uncovered a relationship between HFE status and manganese metabolism by demonstrating that *Hfe^−/−^* knockout mice have reduced levels of blood manganese [13]. This observation validated an epidemiological study of demonstrating human carriers of disease-associated HFE(C282Y) or HFE(H63D) alleles also have lower blood manganese [13].

The HFE(C282Y) and HFE(H63D) variants in the iron regulatory *HFE* gene are the leading cause of adult onset hereditary hemochromatosis (HH), one of the most common genetic diseases in the North American Caucasian population. C282Y and H63D have prevalence in North American populations of 7–17% and 10–32%, respectively [14]. *HFE*-associated HH is the most common Mendelian inherited trait of northern Europeans, with a prevalence of 1∶200 to 1∶500 [15], [16]. Defects in the *HFE* gene promote increased intestinal iron absorption and progressive tissue deposition of the metal resulting in liver damage and disease, congestive heart failure, and premature death. Mice with either the orthologous mutations or null allele display the same iron-loading HH phenotype observed in humans [17].

The effects of iron loading on manganese *in vivo* have been well established [13], [18], [19]. Recent molecular studies have documented a role for divalent metal transporter-1 (DMT1) in manganese uptake [20]–[23]. DMT1 functions in dietary iron absorption across the apical surface of the intestinal mucosa [24], [25] and transports iron from endocytosed transferrin to enable heme synthesis by erythroid cells [26]. Because impaired DMT1 function also results in reduced manganese transport [20], the transporter appears to play an important physiological role in the metabolism of this metal as well. Emerging new evidence indicates that the iron exporter ferroportin (Fpn) [27], [28] also transports manganese [29], [30]. There is strong evidence in the literature that HFE deficiency alters levels of both transporters [31]–[33]. However, while the influence of HFE deficiency on dietary iron absorption has been characterized, its potential effects on manganese metabolism have not been explored. Therefore, we undertook this investigation to characterize the uptake and distribution of the metal in *Hfe* ^−/−^ knockout mice after intravenous, intragastric, and intranasal administration of ^54^Mn.

## Results and Discussion

### Iron loading characteristics of HFE deficiency

**Figure 1:**
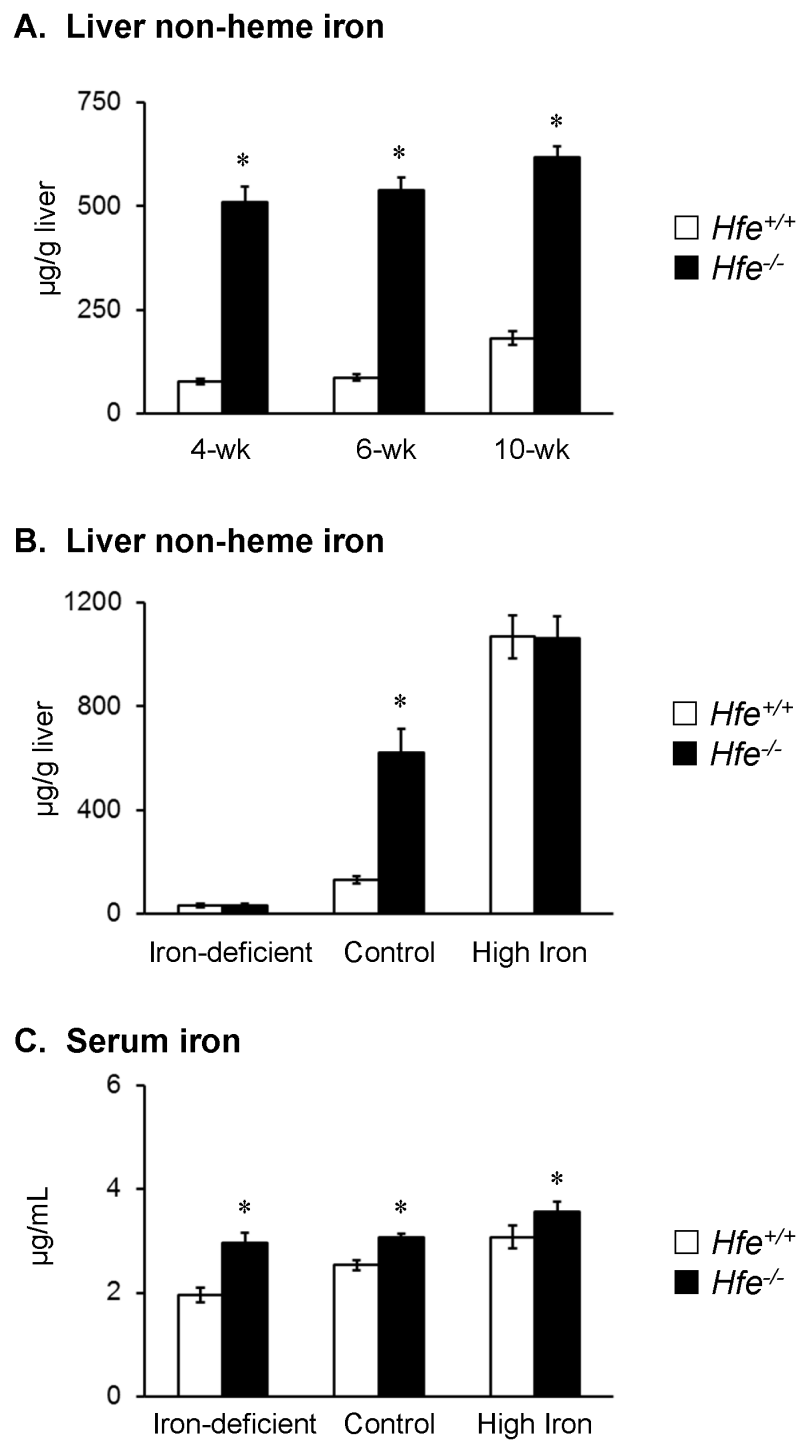
HFE deficiency and iron-loading. Weanling mice were fed facility chow (220 mg/kg iron), euthanized with isoflurane overdose at the indicated ages. Liver non-heme iron levels (**A**) were determined for *Hfe* ^+/+^ (empty bar) and *Hfe* ^−/−^ (closed bar) mice. Data were presented as means ± SEM (n = 9–14 per group). Weanling mice were fed iron-deficient (5 mg/kg iron), control (50 mg/kg), or high iron diet (20,000 mg/kg). After 5 weeks, mice were euthanized with isoflurane overdose, and liver (**B**) and serum non-heme iron (**C**) levels were determined for *Hfe* ^+/+^ and *Hfe* ^−/−^ mice. Data were presented as means ± SEM (n = 6–12 per group). * *P*<0.05 between *Hfe* ^+/+^ and *Hfe* ^−/−^ mice determined by two-way ANOVA followed by Tukey's post-hoc comparison.

Several studies have characterized the iron-loading phenotype of *Hfe* knockout (*Hfe^−/−^*) mice [17], [34]–[36]. In our hands, *Hfe^−/−^* mice also displayed an age-dependent increase in liver non-heme iron levels, which were elevated as early as 4 weeks of age (Figure 1A; *P*<0.001; n = 9–14 per group). These data are consistent with findings by other investigators [37], [38]. To characterize the effect of dietary iron on the iron-loading phenotype, weanling mice were fed iron deficient (5 mg/kg), control (50 mg/kg) or high iron (20,000 mg/kg) diet. Non-heme iron levels in both liver and serum increased in a manner corresponding to dietary iron content regardless of genotype (Figure 1B and C). Liver non-heme iron levels were greater in *Hfe* ^−/−^ mice than in wild-type control (*Hfe* ^+/+^) mice fed control diet (Figure 1B; *P*<0.001; n = 8–12 per group), while the other two diets did not show significant differences between the two strains. Similarly, *Hfe* ^−/−^ mice fed the control diet displayed significantly higher serum iron concentrations compared with *Hfe* ^+/+^ mice (Figure 1C; *P*<0.001; n = 8–12 per group). It is notable that this pattern was also observed in mice fed iron-deficient diet and high iron diet (Figure 1C; *P*<0.001 and *P* = 0.042, respectively; n = 6–8 per group). In contrast to results reported by others [39], [40], *Hfe^−/−^* and *Hfe^+/+^* mice fed a high iron diet had similar liver non-heme iron levels, but this difference could be due to strain variation (129/SvJ *vs* C57BL/6) or the different duration of dietary iron (2–6 weeks) [39]–[41]. To control for the influence of body iron status on the metal uptake studies described below, both *Hfe^−/−^* and *Hfe^+/+^* mice were fed the control diet containing 50 mg/kg iron for 5 weeks after the time of weaning.

### HFE deficiency enhances intestinal uptake of ^59^Fe and clearance of ^59^Fe from circulation

**Figure 2:**
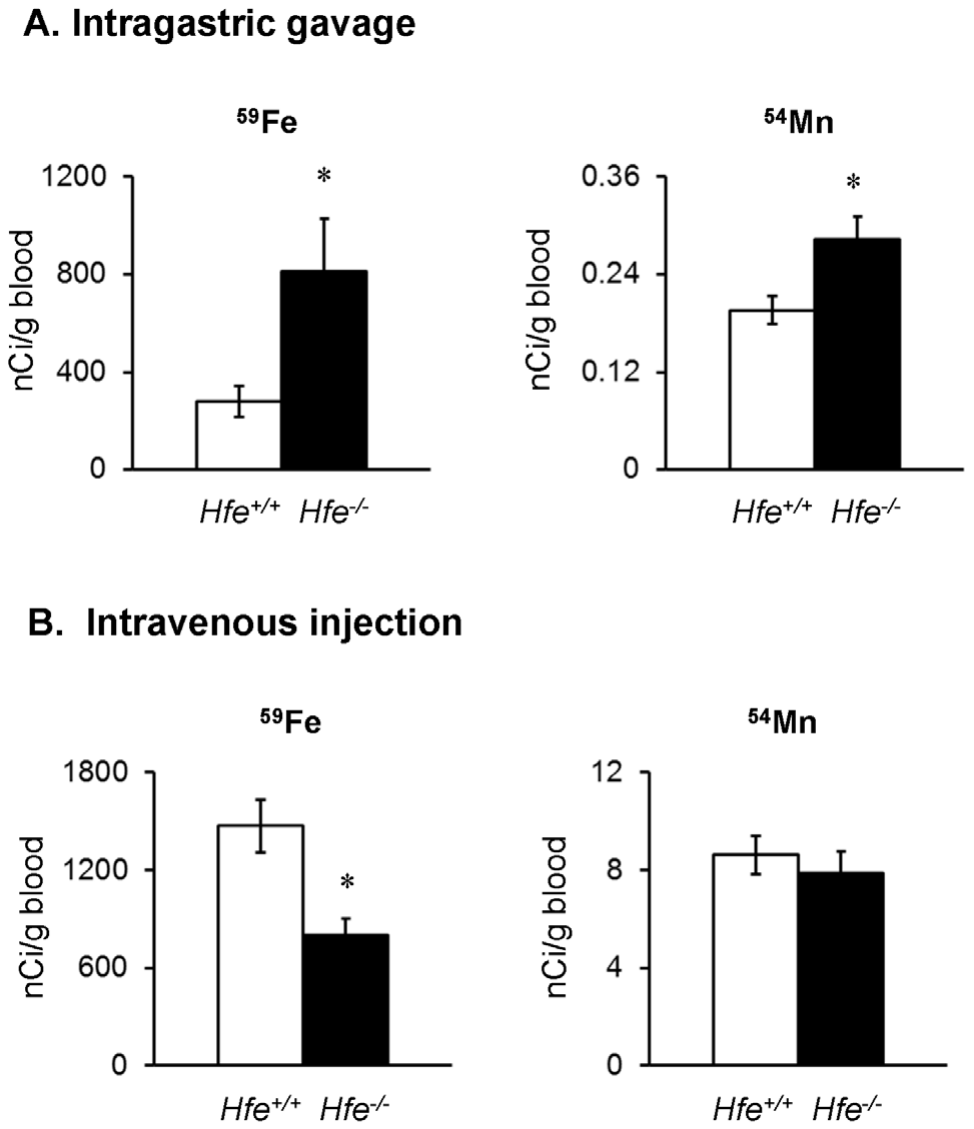
Effect of HFE deficiency on absorption of ^59^Fe and ^54^Mn after intragastric gavage and intravenous injection. Blood levels of ^59^Fe as nCi/g blood were characterized 4 h post-dose of ^59^FeCl_3_ (200 µCi/kg body weight) to mice by intragastric gavage (**A**) and intravenous injection (**B**). Empty and closed bars represent *Hfe* ^+/+^ and *Hfe* ^−/−^ mice, respectively. Data were presented as mean ± SEM (n = 4–5 per group). Blood levels of ^54^Mn as nCi/g blood were characterized 1 h post-dose of ^54^MnCl_2_ (200 µCi/kg body weight) to mice by intragastric gavage (**A**) and intravenous injection (**B**). Data were presented as means ± SEM (n = 4–10 per group). * *P*<0.05 between *Hfe* ^+/+^ and *Hfe* ^−/−^ mice determined by two-sample *t*-test.

To examine the influence of HFE on intestinal iron uptake, ^59^Fe was administered to *Hfe^−/−^* and *Hfe^+/+^* mice by intragastric gavage. Iron was reduced to the ferrous form using freshly dissolved ascorbate immediately prior to instillation. Blood levels of ^59^Fe were determined 4 h after administration (Figure 2A). *Hfe* ^−/−^ mice accumulated a greater amount of iron in blood over the 4-hour period compared to *Hfe^+/+^* wild-type controls (*P* = 0.034; n = 4–5 per group). This observation is consistent with the hyperabsorption of iron from the gut of *Hfe^−/−^* mice [36], [37], [42]. However, we considered the additional possibility that increased appearance of ^59^Fe in the blood after gavage could be due to decreased clearance. Thus, we also characterized blood clearance following intravenous injection of ^59^Fe (Figure 2B). Four hours after intravenous injection, the amount of ^59^Fe in the blood was less in *Hfe^−/−^* compared to *Hfe^+/+^* mice (*P* = 0.008; n = 5 per group), indicating that loss of HFE function contributes to enhanced uptake of iron by peripheral tissues and/or excretion from the body. The accelerated blood clearance promoted by HFE deficiency could be explained by an increased fraction of non-transferrin-bound iron, which displays faster clearance kinetics than transferrin-bound iron [43]. Trinder et al [39] have shown that ^59^FeTf uptake is similar in *Hfe* knockout and wild-type mice. Despite more rapid clearance of injected ^59^Fe, blood ^59^Fe levels after gavage were still greater in *Hfe^−/−^* mice (Figure 2A). These combined data suggest an even larger extent of “intrinsic” intestinal uptake (bioavailability) of iron in the absence of HFE. The calculated bioavailability after correcting for blood clearance of iron during absorption over the 4 hour time period was 3-fold greater in *Hfe* ^−/−^ mice compared with *Hfe* ^+/+^ mice (Table 1, *P*<0.001, n = 4–5 per group). At a mechanistic level, the up-regulation of the intestinal iron transporters DMT1 and/or Fpn has been reported in HFE deficiency [31]–[33] and would contribute to the observed effects as discussed below.

**Table 1:** Intestinal bioavailability of iron and manganese.

	Wild-type (%)			HFE-deficient (%)		
^59^Fe	19.0	±	4.2	81.1*	±	7.3
^54^Mn	2.3	±	0.2	3.6*	±	0.4

Mice were euthanized after intravenous injection or intragastric gavage of the indicated isotopes (200 µCi/kg body weight). Blood and tissues were collected 4-hour after ^59^Fe dose or 1-hour after ^54^Mn dose. Blood concentration after intragastric gavage was divided by the concentration determined after intravenous injection to estimate time-variant intestinal bioavailability of each metal. Data are presented as the mean ± SEM.

* *P*<0.05 between *Hfe* ^+/+^ and *Hfe* ^−/−^ mice determined by two-sample *t*-test.

### HFE deficiency promotes delivery of ^59^Fe to liver and heart

**Figure 3:**
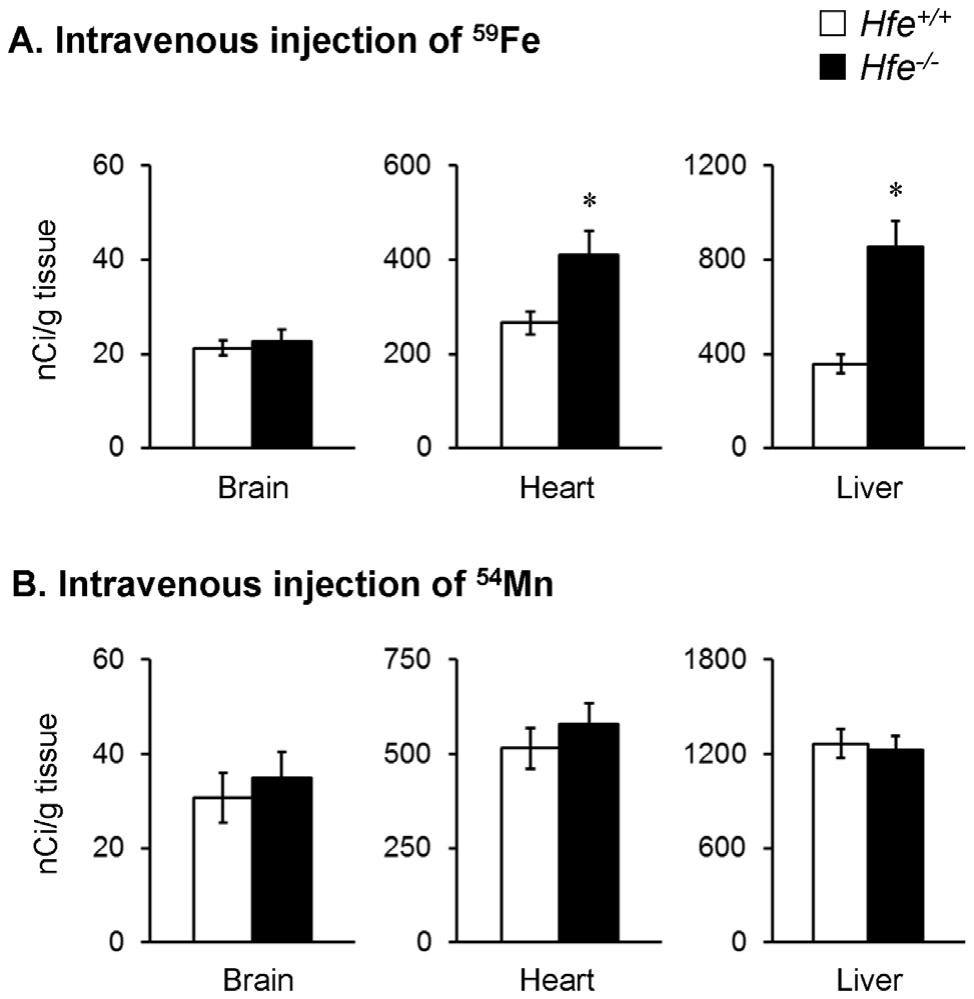
Uptake of injected ^59^Fe and ^54^Mn to brain, heart and liver. Levels of ^59^Fe in brain, heart and liver (as nCi/g tissue) were characterized 4 h post-dose of ^59^FeCl_3_ (200 mCi/kg body weight) to mice by intravenous injection (**A**). Levels of ^54^Mn in brain, heart and liver (as nCi/g tissue) were characterized 1 h post-dose of ^54^MnCl_2_ (200 mCi/kg body weight) to mice by intravenous injection (**B**). Empty and closed bars represent *Hfe* ^+/+^ and *Hfe* ^−/−^ mice, respectively. Data were presented as mean ± SEM (n = 4−5 per group for ^59^Fe and n = 7−8 per group for ^54^Mn). * *P*<0.05 between *Hfe* ^+/+^ and *Hfe* ^−/−^ mice determined by two-sample t-test.

To further examine the influence of HFE on blood clearance of iron, mice were euthanized 4 h after the injected dose and radioactivity in tissue samples was measured to determine the degree of ^59^Fe uptake (Figure 3A). *Hfe* ^−/−^ mice exhibited a 1.4-fold increase in ^59^Fe uptake in the liver compared with *Hfe* ^+/+^ mice (*P* = 0.003; n = 5 per group). Moreover, ^59^Fe in the heart after intravenous injection was 0.5-fold greater in *Hfe* ^−/−^ mice than in *Hfe* ^+/+^ mice (*P* = 0.033; n = 5 per group). Thus, loss of HFE function results in increased tissue uptake to the liver and heart. These observations are of interest since the major causes of death related to iron overload hemochromatosis are liver toxicity and cardiomyopathy [44]. Previous studies by Ajioka et al. [37] reported increased liver distribution of ^59^Fe after gavage. The idea that loss of HFE function leads to hepatic iron loading is consistent with other studies [17], [34]–[36]. Turoczi et al. [45] investigated iron loading of cardiac tissue in *Hfe^−/−^* mice and showed increased iron deposition and reactive oxygen species (ROS). These data support the notion that preferential uptake of iron into these two major organs is associated with metal-related oxidative stress and tissue damage. While the presence of increased non-transferrin bound iron could promote these effects [43], it is possible that differential expression of certain iron transporters contributes to the specific uptake of ^59^Fe; for example, Zip14 in liver [46]. In our experiments, the total radioactivity remaining in the carcass was similar in the two groups (data not shown), suggesting that excretion of iron cannot account for increased blood clearance in HFE deficiency. We conclude that the deposition of iron into key target tissues, particularly the liver, is promoted by HFE deficiency.

### Loss of HFE function increases intestinal manganese uptake

To determine the role of HFE in manganese absorption, we also measured the amount of ^54^Mn in blood after intragastric gavage or intravenous injection of the radioisotope (Figure 2). *Hfe* ^−/−^ mice displayed higher blood ^54^Mn levels than *Hfe* ^+/+^ mice 1 h after gavage (Figure 2A; *P* = 0.036; n = 4 per group), indicating that like iron, intestinal absorption of manganese is enhanced by HFE deficiency (Table 1). Unlike iron, there was no difference in blood clearance of ^54^Mn administered by injection (Figure 2B; *P* = 0.488; n = 7–8 per group). Tissue distribution of ^54^Mn was similar between *Hfe^−/−^* and *Hfe^+/+^* mice with no difference in uptake by heart or liver (Figure 3B). The observations that manganese clearance from circulation is not altered despite high serum iron and accelerated iron clearance in HFE deficiency (Figure 2B) is interesting. It is possible there are potential differences in the kinetics of manganese clearance from circulation by both *Hfe^+/+^* and *Hfe* ^−/−^ mice earlier than the 1 h time point of our tracer study. We chose 1 h for ^54^Mn sampling since manganese absorption reached a plateau level in the brain 1 h following intranasal instillation of ^54^Mn and we therefore monitored blood clearance over the same time frame (see below). Further study is necessary to more fully characterize clearance of manganese from circulation in *Hfe^−/−^* mice, including the effects of continuous administration and chronic inhalation of manganese [9], [47], [48]. Although the kinetics of manganese clearance after intravenous injection need to be better defined, the fact that ^54^Mn did not accumulate in liver and heart of *Hfe^−/−^* mice suggests the removal of manganese from the vasculature may follow pathways that are different from iron.

### Olfactory manganese uptake into the brain is enhanced by HFE deficiency

**Figure 4:**
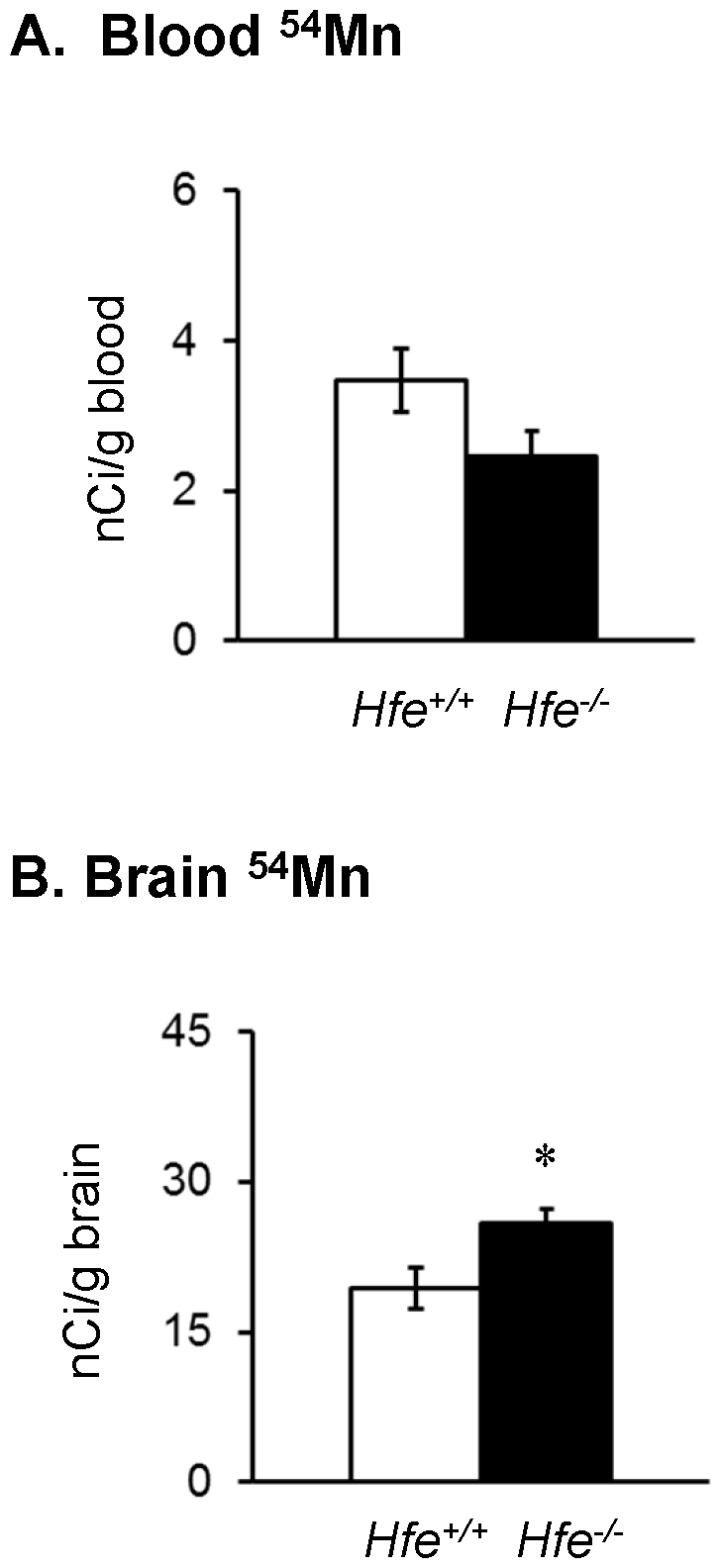
Effect of HFE deficiency on manganese uptake after intranasal instillation. One hour after intranasal instillation of ^54^MnCl_2_ (200 mCi/kg), blood (**A**) and brain (**B**) samples were weighed and radioactivity was measured to calculate isotope level as nCi/g blood or brain. Empty and closed bars represent *Hfe* ^+/+^ and *Hfe* ^−/−^ mice, respectively. Data were presented as means ± SEM (n = 7−8 per group). * *P*<0.05 between *Hfe* ^+/+^ and *Hfe* ^−/−^ mice determined by twosample *t*-test.

Absorption of airborne manganese is an increasing concern due to neurotoxicity of the metal [49]. To study the influence of HFE deficiency on olfactory absorption, uptake of ^54^MnCl_2_ was examined in *Hfe^−/−^* and *Hfe^+/+^* mice after intranasal instillation. Preliminary studies demonstrated that ^54^Mn absorption to the brain was maximal 1 h post-instillation. Levels of ^54^Mn in *Hfe^−/−^* and *Hfe^+/+^* in the blood 1 h after instillation did not differ (Figure 4A; *P* = 0.090; n = 7–8 per group). It is interesting to note that the overall amount of isotope absorbed from the nasal cavity into the blood was much higher than the amount taken up after intragastric gavage, regardless of genotype (Figure 2A). These data demonstrate the greater efficiency of systemic manganese uptake *via* the olfactory pathway compared to the oral route [7]–[9], confirming that inhalation is a critical route of manganese intoxication, especially in occupational settings [5], [6]. Moreover, our data show that uptake to the brain after intranasal instillation was increased in *Hfe* ^−/−^ mice compared with *Hfe* ^+/+^ mice (Figure 4B; *P* = 0.028; n = 7–8 per group). This effect appears to be specific for brain uptake by the olfactory pathway since no differences were detected between the groups after intragastric gavage or intravenous injection (Figure 2 and 3B). Notably, the uptake of manganese (Figure 3B) and iron (Figure 3A) across the blood-brain barrier in HFE deficiency is unaffected. Combined, these observations suggest different mechanism(s) of metal uptake between olfactory and vascular transport to the brain. The latter may be primarily mediated by transferrin. Nonetheless, the observed increase in uptake to the brain of *Hfe^−/−^* mice suggests that individuals with HFE-associated hemochromatosis may be more vulnerable to neurotoxicity of inhaled manganese.

### DMT1 and Fpn expression in olfactory bulb

**Figure 5:**
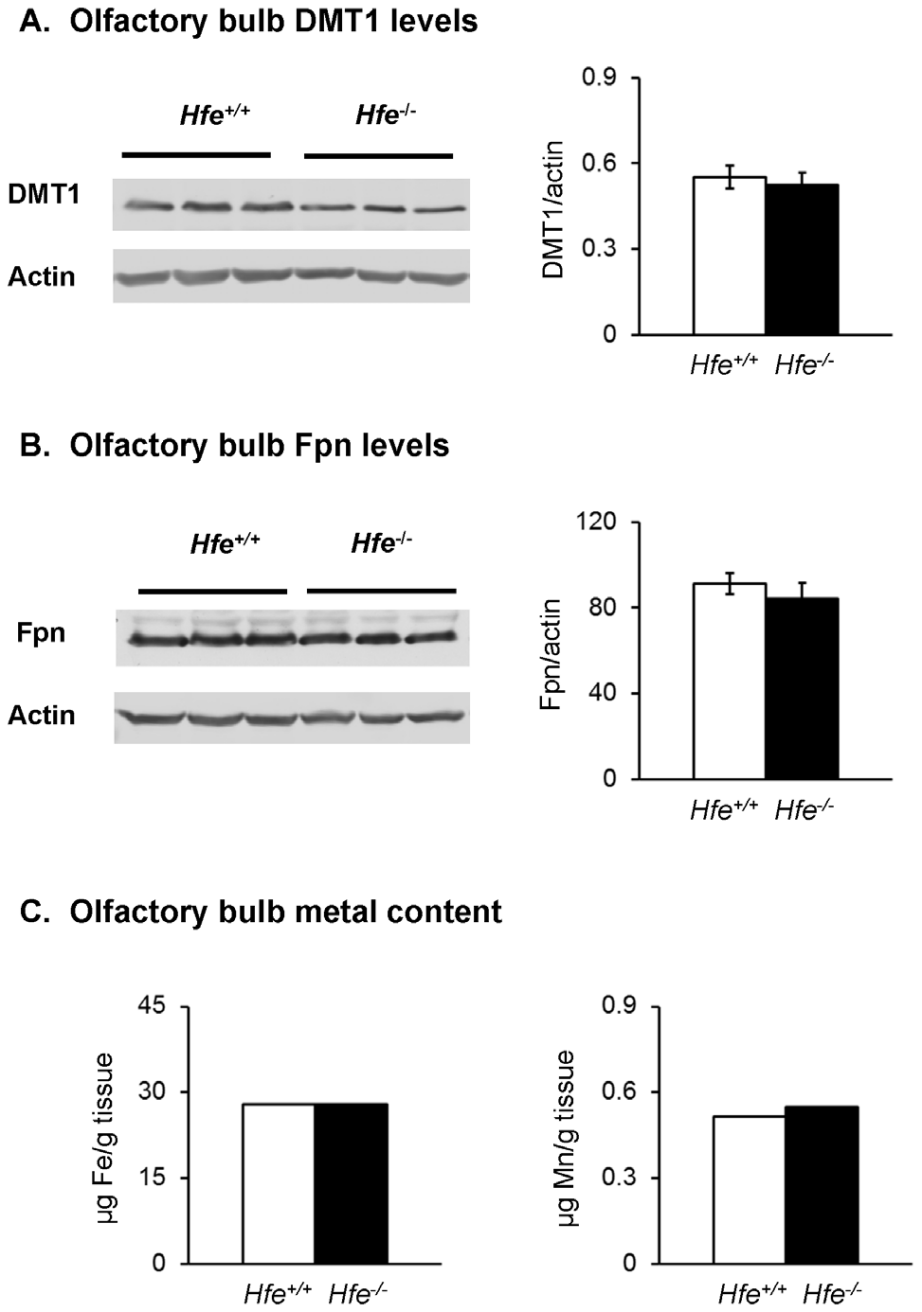
Levels of transporters and metals in olfactory bulbs of wild-type and HFE knockout mice. Weanling mice were fed control diet and euthanized to collect olfactory bulbs. Levels of DMT1 (**A**) and Fpn (**B**) were determined by western blot. Data were presented as means ± SEM (n = 6 per group). Olfactory bulbs from 3 male and 5 female *Hfe* ^+/+^ mice and 4 male and 4 female *Hfe* ^−/−^ mice were pooled and determined for iron and manganese levels by ICP-MS (**C**). Data were presented as means.

It is known that increased absorption of iron across the intestinal mucosa is promoted by up-regulation of DMT1 and Fpn [24], [28], [50]. Both transporters are post-transcriptionally regulated by iron-responsive elements [51], [52]. In the case of HFE deficiency, post-translational modification also is imparted by the iron regulatory hormone hepcidin [53], [54], which is inappropriately down-regulated to enhance Fpn levels [55], [56]. We have previously found that systemic iron deficiency induces DMT1 up-regulation in the olfactory epithelium [20] and olfactory bulbs [57], an effect that is associated with increased olfactory transport of manganese to the brain [20], [21]. Therefore, we compared DMT1 and ferroportin levels in the olfactory bulbs from *Hfe^−/−^* with *Hfe^+/+^* mice (Figure 5A and B). Despite the iron overload status of *Hfe^−/−^* mice (Figure 1), levels of both transporters were similar in the two groups. However, ICP-MS analysis of the metal content of the olfactory bulbs from wild-type and knockout mice showed no differences that would otherwise promote changes in the expression pattern of the transporters at the tissue level (Figure 5C). Levels of iron were 50-fold greater than manganese. These results indicate that there could be other factors independent of DMT1 or Fpn involved in manganese transport across nasal cavity into brain that are affected by the loss of HFE function. For example, local regulation of hepcidin may play an important role in metal uptake in the brain [58]. Other transport pathways could also influence brain uptake of airborne manganese, including newly emerging roles for SLC30A10 transporters [59], [60] and manganese binding proteins like calprotectin [61]. Further work is necessary to define the manganese pathways affected in *Hfe^−/−^* mice and the functional consequences due to the increased uptake of manganese from the nasal cavity to the brain.

## Conclusions

- HFE-deficient mice displayed elevated intestinal absorption of both iron and manganese.
- HFE-deficient mice had greater uptake of intravenously injected ^59^Fe by liver and heart, while uptake of ^54^Mn to these tissues was unaffected.
- Regardless of genotype, greater levels of ^54^Mn were present in the blood after intranasal instillation compared to administration by intragastric gavage.
- HFE-deficient mice had greater uptake of intranasally-instilled ^54^Mn into the brain.
- DMT1 and Fpn are expressed in olfactory bulb, however, levels of both transporters were similar in HFE-deficient and wild-type mice.
- HFE deficiency was not associated with altered levels of iron or manganese in olfactory bulbs; levels of iron were 50-fold greater than manganese.
- Loss of HFE increases uptake of manganese to the brain by the olfactory pathway, suggesting individuals with HFE-associated hemochromatosis may be at greater risk for inhalation exposures.

## Materials and Methods

### Ethics statement

This study was performed in strict accordance with the recommendations in the Guide for the Care and Use of Laboratory Animals of the National Institutes of Health. The protocol was approved by the Harvard Medical Area Animal Care and Use Committee (Animal Experimentation Protocol AEP #04545 and #04692).

### Animal care and procedures

HFE-deficient (*Hfe* ^−/−^) mice and wild-type control (*Hfe* ^+/+^) mice were kindly provided by Dr. Nancy Andrews (Duke University, NC). All mice used for these studies were on the 129S6/SvEvTac background. Weanling mice were fed a diet containing 50 mg iron/kg (as ferric citrate, TD07800, Harlan Teklad) for 5 weeks. For experiments testing the effect of dietary iron, groups of knockout and wild-type mice were also placed on iron-deficient diet (5 mg iron/kg diet; TD99397), or iron-overload diet (20,000 mg/kg; as carbonyl iron, TD08714). Mice were euthanized under isoflurane inhalation (5%) to obtain serum and liver, and non-heme iron concentrations in these tissues were determined by spectrophotometric methods [62], [63]. All experiments were carried out between 12–3 p.m. to avoid circadian effects on iron metabolism. No obvious gender differences were noted in the transport studies described below.

To characterize gastrointestinal absorption of iron, 8-week-old mice were fasted for 4 hours, anesthetized with up to 2% vaporized isoflurane, and administered ^59^FeCl_3_ (200 µCi/kg body weight) or ^54^MnCl_2_ (200 µCi/kg body weight) by intragastric gavage using a 20 gauge 1.5-inch gavage needle. ^59^Fe was diluted in Tris-buffered saline containing ascorbic acid (10 mM) at 1.5 mL/kg immediately prior to administration; ^54^Mn was diluted in phosphate-buffered saline (PBS). Mice were euthanized by isoflurane overdose 4 h post-gavage to collect blood *via* the inferior vena cava and tissues were dissected. Radioactivity was determined using a WIZARD 1410 gamma counter (Perkin Elmer). Data were analyzed and expressed as nCi/g tissue. Since the blood levels of ^59^Fe represent the amount absorbed from the gut as well as the amount cleared from the circulation, similar cohorts of mice were intravenously injected with the same dose of ^59^Fe or ^54^Mn *via* the tail vein to account for the contribution of clearance to the blood level. To study intranasal absorption, ^54^Mn was instilled into the right nostril at 0.02 mL/kg using a thin polyurethane catheter (mouse jugular catheter; Alzet, Cupertino, CA). Since a pilot study showed that a plateau level of ^54^Mn was achieved in the brain one hour post-instillation, intravenous, intragastric, or intranasal absorption were each studied 1 h post-administration.

### Western blots

Olfactory bulbs from male *Hfe* ^+/+^ and *Hfe* ^−/−^ mice were homogenized in 10 mM Tris, pH 7.4, 150 mM NaCl, 1.0 mM EDTA, 0.1% SDS, 1.0% Triton X-100, 1.0% sodium deoxycholate containing protease inhibitors (Complete Mini, Roche). Aliquots 30 µg (DMT1) or 40 µg (ferroportin) were prepared in sample buffer without boiling and electrophoresed on 10% gels. After transfer to nitrocellulose membranes, blots were blocked with 5% non-fat milk and immunoblotted using rabbit anti-DMT1 (all four isoforms) antibody (1∶1,000; a kind gift from Dr. Jerry Kaplan, The University of Utah, Salt Lake City, UT) or rabbit anti-ferroportin antibody (1∶500; Alpha Diagnostics). Blots were probed with mouse anti-actin (MP Biomedicals) as a loading control. Secondary antibodies were donkey anti-rabbit IRDye800 (DMT1; LI-COR), donkey anti-rabbit HRP (ferroportin; GE) or donkey anti-mouse IRDye680 (Actin; LI-COR). Immunoreactivity was detected using an Odyssey Infrared Imaging System (DMT1 and actin; LI-COR) or ECL (ferroportin; West Pico, Thermo Scientific). Relative intensities of protein bands were determined using Odyssey (version 2.1; LI-COR) or Image J software (version 1.43; NIH).

### ICP-MS

Olfactory bulbs from 3 male and 5 female *Hfe* ^+/+^ mice and 4 male and 4 female *Hfe* ^−/−^ mice were pooled and analyzed for iron and manganese levels by ICP-MS (Trace Element Analysis Laboratory, Dept. of Earth Sciences, Dartmouth College, Hanover, NH).

### Statistical analyses

Values reported were expressed as means ± SEM. For multi-group comparison (gene and diet effect), two-way ANOVA test followed by Tukey's post-hoc comparison was used. Two-sample *t*-test was employed to compare the parameters between *Hfe* ^−/−^ and *Hfe* ^+/+^ mice using Systat 13 (Systat). Differences were considered significant at *P*<0.05.

## References

[1] Yamada M, Ohno S, Okayasu I, Okeda R, Hatakeyama S, et al (1986) Chronic manganese poisoning: a neuropathological study with determination of manganese distribution in the brain. Acta Neuropathol 70: 273–278. 3766127 10.1007/BF00686083

[2] Lucchini R, Bergamaschi E, Smargiassi A, Festa D, Apostoli P (1997) Motor function, olfactory threshold, and hematological indices in manganese-exposed ferroalloy workers. Environ Res 73: 175–180. 9311544 10.1006/enrs.1997.3702

[3] Tran TT, Chowanadisai W, Lonnerdal B, Le L, Parker M, et al (2002) Effects of neonatal dietary manganese exposure on brain dopamine levels and neurocognitive functions. Neurotoxicology 23: 645–651. 12428736 10.1016/s0161-813x(02)00068-2

[4] Bonilla E (1984) Chronic manganese intake induces changes in the motor activity of rats. Exp Neurol 84: 696–700. 6723889 10.1016/0014-4886(84)90216-4

[5] Cotzias GC (1958) Manganese in health and disease. Physiol Rev 38: 503–532. 13567045 10.1152/physrev.1958.38.3.503

[6] Bertinchamps AJ, Miller ST, Cotzias GC (1966) Interdependence of routes excreting manganese. Am J Physiol 211: 217–224. 5911041 10.1152/ajplegacy.1966.211.1.217

[7] Tjalve H, Henriksson J, Tallkvist J, Larsson BS, Lindquist NG (1996) Uptake of manganese and cadmium from the nasal mucosa into the central nervous system via olfactory pathways in rats. Pharmacol Toxicol 79: 347–356. 9000264 10.1111/j.1600-0773.1996.tb00021.x

[8] Brenneman KA, Wong BA, Buccellato MA, Costa ER, Gross EA, et al (2000) Direct olfactory transport of inhaled manganese ((54)MnCl(2)) to the rat brain: toxicokinetic investigations in a unilateral nasal occlusion model. Toxicol Appl Pharmacol 169: 238–248. 11133346 10.1006/taap.2000.9073

[9] Nong A, Teeguarden JG, Clewell HJ 3rd, Dorman DC, Andersen ME (2008) Pharmacokinetic modeling of manganese in the rat IV: Assessing factors that contribute to brain accumulation during inhalation exposure. J Toxicol Environ Health A 71: 413–426. 18306088 10.1080/15287390701838697

[10] Barbeau A (1984) Manganese and extrapyramidal disorders (a critical review and tribute to Dr. George C. Cotzias). Neurotoxicology 5: 13–35. 6538948

[11] Donaldson J (1987) The physiopathologic significance of manganese in brain: its relation to schizophrenia and neurodegenerative disorders. Neurotoxicology 8: 451–462. 3309736

[12] Haynes EN, Heckel P, Ryan P, Roda S, Leung YK, et al (2010) Environmental manganese exposure in residents living near a ferromanganese refinery in Southeast Ohio: a pilot study. Neurotoxicology 31: 468–474. 19879291 10.1016/j.neuro.2009.10.011 PMC2891785

[13] Claus Henn B, Kim J, Wessling-Resnick M, Tellez-Rojo MM, Jayawardene I, et al (2011) Associations of iron metabolism genes with blood manganese levels: a population-based study with validation data from animal models. Environ Health 10: 97. 22074419 10.1186/1476-069X-10-97 PMC3248860

[14] Bradley LA, Johnson DD, Palomaki GE, Haddow JE, Robertson NH, et al (1998) Hereditary haemochromatosis mutation frequencies in the general population. J Med Screen 5: 34–36. 9575458 10.1136/jms.5.1.34

[15] Bacon BR, Powell LW, Adams PC, Kresina TF, Hoofnagle JH (1999) Molecular medicine and hemochromatosis: at the crossroads. Gastroenterology 116: 193–207. 9869618 10.1016/s0016-5085(99)70244-1

[16] Merryweather-Clarke AT, Pointon JJ, Jouanolle AM, Rochette J, Robson KJ (2000) Geography of HFE C282Y and H63D mutations. Genet Test 4: 183–198. 10953959 10.1089/10906570050114902

[17] Levy JE, Montross LK, Cohen DE, Fleming MD, Andrews NC (1999) The C282Y mutation causing hereditary hemochromatosis does not produce a null allele. Blood 94: 9–11. 10381492

[18] Thompson K, Molina R, Donaghey T, Brain JD, Wessling-Resnick M (2006) The influence of high iron diet on rat lung manganese absorption. Toxicol Appl Pharmacol 210: 17–23. 15993455 10.1016/j.taap.2005.05.014

[19] Chua AC, Morgan EH (1996) Effects of iron deficiency and iron overload on manganese uptake and deposition in the brain and other organs of the rat. Biological trace element research 55: 39–54. 8971353 10.1007/BF02784167

[20] Thompson K, Molina RM, Donaghey T, Schwob JE, Brain JD, et al (2007) Olfactory uptake of manganese requires DMT1 and is enhanced by anemia. FASEB J 21: 223–230. 17116743 10.1096/fj.06-6710com PMC2432183

[21] Kim J, Li Y, Buckett PD, Bohlke M, Thompson KJ, et al (2012) Iron-responsive olfactory uptake of manganese improves motor function deficits associated with iron deficiency. PLoS One 7: e33533. 22479410 10.1371/journal.pone.0033533 PMC3316579

[22] Roth JA, Garrick MD (2003) Iron interactions and other biological reactions mediating the physiological and toxic actions of manganese. Biochem Pharmacol 66: 1–13. 12818360 10.1016/s0006-2952(03)00145-x

[23] Illing AC, Shawki A, Cunningham CL, Mackenzie B (2012) Substrate profile and metal-ion selectivity of human divalent metal-ion transporter-1. J Biol Chem 287: 30485–30496. 22736759 10.1074/jbc.M112.364208 PMC3436370

[24] Fleming MD, Trenor CC 3rd, Su MA, Foernzler D, Beier DR, et al (1997) Microcytic anaemia mice have a mutation in Nramp2, a candidate iron transporter gene. Nat Genet 16: 383–386. 9241278 10.1038/ng0897-383

[25] Gunshin H, Mackenzie B, Berger UV, Gunshin Y, Romero MF, et al (1997) Cloning and characterization of a mammalian proton-coupled metal-ion transporter. Nature 388: 482–488. 9242408 10.1038/41343

[26] Fleming MD, Romano MA, Su MA, Garrick LM, Garrick MD, et al (1998) Nramp2 is mutated in the anemic Belgrade (b) rat: evidence of a role for Nramp2 in endosomal iron transport. Proc Natl Acad Sci U S A 95: 1148–1153. 9448300 10.1073/pnas.95.3.1148 PMC18702

[27] Abboud S, Haile DJ (2000) A novel mammalian iron-regulated protein involved in intracellular iron metabolism. J Biol Chem 275: 19906–19912. 10747949 10.1074/jbc.M000713200

[28] McKie AT, Marciani P, Rolfs A, Brennan K, Wehr K, et al (2000) A novel duodenal iron-regulated transporter, IREG1, implicated in the basolateral transfer of iron to the circulation. Mol Cell 5: 299–309. 10882071 10.1016/s1097-2765(00)80425-6

[29] Madejczyk MS, Ballatori N (2012) The iron transporter ferroportin can also function as a manganese exporter. Biochim Biophys Acta 1818: 651–657. 22178646 10.1016/j.bbamem.2011.12.002 PMC5695046

[30] Yin Z, Jiang H, Lee ES, Ni M, Erikson KM, et al (2010) Ferroportin is a manganese-responsive protein that decreases manganese cytotoxicity and accumulation. J Neurochem 112: 1190–1198. 20002294 10.1111/j.1471-4159.2009.06534.x PMC2819584

[31] Zoller H, Koch RO, Theurl I, Obrist P, Pietrangelo A, et al (2001) Expression of the duodenal iron transporters divalent-metal transporter 1 and ferroportin 1 in iron deficiency and iron overload. Gastroenterology 120: 1412–1419. 11313311 10.1053/gast.2001.24033

[32] Zoller H, Pietrangelo A, Vogel W, Weiss G (1999) Duodenal metal-transporter (DMT-1, NRAMP-2) expression in patients with hereditary haemochromatosis. Lancet 353: 2120–2123. 10382697 10.1016/S0140-6736(98)11179-0

[33] Rolfs A, Bonkovsky HL, Kohlroser JG, McNeal K, Sharma A, et al (2002) Intestinal expression of genes involved in iron absorption in humans. Am J Physiol Gastrointest Liver Physiol 282: G598–607. 11897618 10.1152/ajpgi.00371.2001

[34] Zhou XY, Tomatsu S, Fleming RE, Parkkila S, Waheed A, et al (1998) HFE gene knockout produces mouse model of hereditary hemochromatosis. Proc Natl Acad Sci U S A 95: 2492–2497. 9482913 10.1073/pnas.95.5.2492 PMC19387

[35] Herrmann T, Muckenthaler M, van der Hoeven F, Brennan K, Gehrke SG, et al (2004) Iron overload in adult Hfe-deficient mice independent of changes in the steady-state expression of the duodenal iron transporters DMT1 and Ireg1/ferroportin. J Mol Med 82: 39–48. 14618243 10.1007/s00109-003-0508-x

[36] Bahram S, Gilfillan S, Kuhn LC, Moret R, Schulze JB, et al (1999) Experimental hemochromatosis due to MHC class I HFE deficiency: immune status and iron metabolism. Proc Natl Acad Sci U S A 96: 13312–13317. 10557317 10.1073/pnas.96.23.13312 PMC23944

[37] Ajioka RS, Levy JE, Andrews NC, Kushner JP (2002) Regulation of iron absorption in Hfe mutant mice. Blood 100: 1465–1469. 12149232 10.1182/blood-2001-11-0037

[38] Rodrigues P, Lopes C, Mascarenhas C, Arosio P, Porto G, et al (2006) Comparative study between Hfe−/− and beta2m−/− mice: progression with age of iron status and liver pathology. Int J Exp Pathol 87: 317–324. 16875497 10.1111/j.1365-2613.2006.00491.x PMC2517374

[39] Trinder D, Olynyk JK, Sly WS, Morgan EH (2002) Iron uptake from plasma transferrin by the duodenum is impaired in the Hfe knockout mouse. Proc Natl Acad Sci U S A 99: 5622–5626. 11943867 10.1073/pnas.082112299 PMC122820

[40] Simpson RJ, Debnam E, Beaumont N, Bahram S, Schumann K, et al (2003) Duodenal mucosal reductase in wild-type and Hfe knockout mice on iron adequate, iron deficient, and iron rich feeding. Gut 52: 510–513. 12631660 10.1136/gut.52.4.510 PMC1773615

[41] Dupic F, Fruchon S, Bensaid M, Loreal O, Brissot P, et al (2002) Duodenal mRNA expression of iron related genes in response to iron loading and iron deficiency in four strains of mice. Gut 51: 648–653. 12377801 10.1136/gut.51.5.648 PMC1773425

[42] Griffiths WJ, Sly WS, Cox TM (2001) Intestinal iron uptake determined by divalent metal transporter is enhanced in HFE-deficient mice with hemochromatosis. Gastroenterology 120: 1420–1429. 11313312 10.1053/gast.2001.24050

[43] Craven CM, Alexander J, Eldridge M, Kushner JP, Bernstein S, et al (1987) Tissue distribution and clearance kinetics of non-transferrin-bound iron in the hypotransferrinemic mouse: a rodent model for hemochromatosis. Proc Natl Acad Sci U S A 84: 3457–3461. 3472216 10.1073/pnas.84.10.3457 PMC304890

[44] Pietrangelo A (2004) Hereditary hemochromatosis–a new look at an old disease. N Engl J Med 350: 2383–2397. 15175440 10.1056/NEJMra031573

[45] Turoczi T, Jun L, Cordis G, Morris JE, Maulik N, et al (2003) HFE mutation and dietary iron content interact to increase ischemia/reperfusion injury of the heart in mice. Circ Res 92: 1240–1246. 12750309 10.1161/01.RES.0000076890.59807.23

[46] Nam H, Wang CY, Zhang L, Zhang W, Hojyo S, et al (2013) ZIP14 and DMT1 in the liver, pancreas, and heart are differentially regulated by iron deficiency and overload: implications for tissue iron uptake in iron-related disorders. Haematologica 10.3324/haematol.2012.072314 PMC3696608 23349308

[47] Andersen ME, Gearhart JM, Clewell HJ 3rd (1999) Pharmacokinetic data needs to support risk assessments for inhaled and ingested manganese. Neurotoxicology 20: 161–171. 10385880

[48] Dorman DC, Brenneman KA, McElveen AM, Lynch SE, Roberts KC, et al (2002) Olfactory transport: a direct route of delivery of inhaled manganese phosphate to the rat brain. J Toxicol Environ Health A 65: 1493–1511. 12396865 10.1080/00984100290071630

[49] Roels HA, Bowler RM, Kim Y, Claus Henn B, Mergler D, et al (2012) Manganese exposure and cognitive deficits: a growing concern for manganese neurotoxicity. Neurotoxicology 33: 872–880. 22498092 10.1016/j.neuro.2012.03.009 PMC3839941

[50] Canonne-Hergaux F, Gruenheid S, Ponka P, Gros P (1999) Cellular and subcellular localization of the Nramp2 iron transporter in the intestinal brush border and regulation by dietary iron. Blood 93: 4406–4417. 10361139

[51] Lee PL, Gelbart T, West C, Halloran C, Beutler E (1998) The human Nramp2 gene: characterization of the gene structure, alternative splicing, promoter region and polymorphisms. Blood Cells Mol Dis 24: 199–215. 9642100 10.1006/bcmd.1998.0186

[52] Montosi G, Donovan A, Totaro A, Garuti C, Pignatti E, et al (2001) Autosomal-dominant hemochromatosis is associated with a mutation in the ferroportin (SLC11A3) gene. J Clin Invest 108: 619–623. 11518736 10.1172/JCI13468 PMC209405

[53] Park CH, Valore EV, Waring AJ, Ganz T (2001) Hepcidin, a urinary antimicrobial peptide synthesized in the liver. J Biol Chem 276: 7806–7810. 11113131 10.1074/jbc.M008922200

[54] Nemeth E, Tuttle MS, Powelson J, Vaughn MB, Donovan A, et al (2004) Hepcidin regulates cellular iron efflux by binding to ferroportin and inducing its internalization. Science 306: 2090–2093. 15514116 10.1126/science.1104742

[55] Bridle KR, Frazer DM, Wilkins SJ, Dixon JL, Purdie DM, et al (2003) Disrupted hepcidin regulation in HFE-associated haemochromatosis and the liver as a regulator of body iron homoeostasis. Lancet 361: 669–673. 12606179 10.1016/S0140-6736(03)12602-5

[56] Muckenthaler M, Roy CN, Custodio AO, Minana B, deGraaf J, et al (2003) Regulatory defects in liver and intestine implicate abnormal hepcidin and Cybrd1 expression in mouse hemochromatosis. Nat Genet 34: 102–107. 12704390 10.1038/ng1152

[57] Ruvin Kumara VM, Wessling-Resnick M (2012) Olfactory ferric and ferrous iron absorption in iron-deficient rats. Am J Physiol Lung Cell Mol Physiol 302: L1280–1286. 22492739 10.1152/ajplung.00004.2012 PMC3379048

[58] Li L, Holscher C, Chen BB, Zhang ZF, Liu YZ (2011) Hepcidin treatment modulates the expression of divalent metal transporter-1, ceruloplasmin, and ferroportin-1 in the rat cerebral cortex and hippocampus. Biol Trace Elem Res 143: 1581–1593. 21274654 10.1007/s12011-011-8967-3

[59] Tuschl K, Clayton PT, Gospe SM Jr, Gulab S, Ibrahim S, et al (2012) Syndrome of hepatic cirrhosis, dystonia, polycythemia, and hypermanganesemia caused by mutations in SLC30A10, a manganese transporter in man. Am J Hum Genet 90: 457–466. 22341972 10.1016/j.ajhg.2012.01.018 PMC3309187

[60] Quadri M, Federico A, Zhao T, Breedveld GJ, Battisti C, et al (2012) Mutations in SLC30A10 cause parkinsonism and dystonia with hypermanganesemia, polycythemia, and chronic liver disease. Am J Hum Genet 90: 467–477. 22341971 10.1016/j.ajhg.2012.01.017 PMC3309204

[61] Damo SM, Kehl-Fie TE, Sugitani N, Holt ME, Rathi S, et al (2013) Molecular basis for manganese sequestration by calprotectin and roles in the innate immune response to invading bacterial pathogens. Proc Natl Acad Sci U S A 110: 3841–3846. 23431180 10.1073/pnas.1220341110 PMC3593839

[62] Kim J, Molina RM, Donaghey TC, Buckett PD, Brain JD, et al (2011) Influence of DMT1 and iron status on inflammatory responses in the lung. Am J Physiol Lung Cell Mol Physiol 300: L659–665. 21278260 10.1152/ajplung.00343.2010 PMC3075097

[63] Chaudhury C, Kim J, Mehnaz S, Wani MA, Oberyszyn TM, et al (2006) Accelerated transferrin degradation in HFE-deficient mice is associated with increased transferrin saturation. J Nutr 136: 2993–2998. 17116709 10.1093/jn/136.12.2993

